# Residence and insurance inequities in the timeliness of access to first-eye cataract surgery in Eastern China

**DOI:** 10.1007/s10792-026-04069-9

**Published:** 2026-04-06

**Authors:** Chen Qin, Jie Zhu, Tian Yang

**Affiliations:** Department of Ophthalmology, Northern Jiangsu People’s Hospital Affiliated to Yangzhou University, 98 Nantong West Road, Yangzhou, 225001 Jiangsu China

**Keywords:** Cataract surgery, Health equity, Electronic health records, Timeliness, Best-corrected visual acuity, Insurance

## Abstract

**Purpose:**

To quantify residence and insurance inequities in the timeliness of access to first-eye cataract surgery in Eastern China, using better-eye best-corrected visual acuity (BCVA) at surgical-pathway entry as an electronic health record–derived indicator.

**Methods:**

We conducted a retrospective cohort study of consecutive first-eye cataract surgeries at a tertiary hospital in Yangzhou, China (August 2024–July 2025). Analyses were restricted to local insured residents; non-local and self-pay patients were excluded. The primary outcome was better-eye BCVA (logMAR), defined as the better (lower logMAR) value between the two eyes measured at the admission-registration (pathway-entry) assessment. Adjusted differences were estimated using multivariable linear regression with surgeon fixed effects and heteroskedasticity-consistent (HC3) robust standard errors. Prespecified categorical analyses used multinomial logistic regression.

**Results:**

Among 1438 eligible patients, bilateral BCVA was measured at pathway entry in 1437 (99.9%). Compared with Urban–Employee patients, adjusted better-eye BCVA was worse in Urban–Resident (0.09 logMAR; 95% CI 0.01–0.17), Rural–Employee (0.11; 0.01–0.21), and Rural–Resident patients (0.17; 0.11–0.24); adjusted marginal means were 0.32, 0.41, 0.43, and 0.49 logMAR, respectively. Rural–Resident patients were less likely to enter in the early category (RRR 0.52; 95% CI 0.39–0.69) and more likely to enter in the late category (RRR 1.76; 95% CI 1.15–2.69).

**Conclusion:**

Residence and insurance inequities in the timeliness of access to first-eye cataract surgery were evident. Better-eye BCVA at pathway entry is a low-cost, scalable metric that can complement coverage and postoperative outcomes to guide pathway and financing interventions.

**Supplementary Information:**

The online version contains supplementary material available at 10.1007/s10792-026-04069-9.

## Background

Cataract remains a leading cause of blindness and vision impairment worldwide, and cataract surgery is among the most cost-effective interventions in modern medicine [1–3]. As cataract services expand, evaluation has moved beyond surgical volume toward whether those in need receive surgery, achieve good outcomes, and do so equitably across population groups [1, 2]. “Effective cataract surgical coverage” (eCSC) reflects this shift by integrating coverage with postoperative visual outcomes and has been proposed for universal health coverage monitoring [4, 5]. In practice, eCSC and related approaches facilitate monitoring not only of service delivery, but also of benefit and equity [6–8].

However, effective coverage metrics leave an important blind spot: timeliness. Two systems can achieve similar coverage and postoperative outcomes while differing substantially in when patients receive surgery along their functional trajectory. Timeliness is a core domain of health-care quality, and avoidable delays may convert remediable impairment into disability and loss of independence, particularly among older adults [9–11].

Delays are not merely administrative frictions. Waiting for cataract surgery has been associated with further vision loss and reduced quality of life during the waiting period [12]. Evidence from trials and meta-analyses also suggests that first-eye cataract surgery may reduce falls in older adults, supporting a plausible pathway by which delayed restoration of binocular reserve could contribute to mobility-related harm [13, 14]. Although downstream functional outcomes were not measured in the present study, these findings motivate pragmatic system metrics that can detect timeliness gaps and inequities upstream of potentially avoidable functional decline.

For routine monitoring, timeliness indicators must be scalable and available in real-world data systems. Cataract decision-making should remain individualized and function-driven rather than dictated by a single acuity cutoff; nevertheless, preoperative visual acuity is one of the few routinely captured data elements widely available across electronic health records (EHRs) and administrative systems [15–19]. For first-eye cataract surgery, a binocularly anchored measure is particularly relevant because everyday functioning often depends on the better-seeing eye. In routine EHR environments where patient-reported visual function and standardized cataract grading are rarely captured at scale, preoperative better-eye best-corrected visual acuity (BCVA) measured at routine preoperative assessment provides an interpretable proxy for binocular functional reserve when patients enter the surgical pathway [15].

Importantly, the distribution of preoperative acuity among treated patients can be interpreted as a “revealed” timing-of-access pattern shaped by care-seeking, referral processes, pathway frictions, and financing rather than a biologically fixed threshold [6, 7]. Consistent with this interpretation, registry and cross-country analyses have documented substantial variation and temporal shifts in preoperative better-eye acuity distributions, indicating that health-system context influences when patients receive surgery [16–18, 20]. Equity gradients have also been reported in mature systems, including worse impairment at first-eye surgery among more deprived groups and inequities in waiting times [21–23]. In this study, worse better-eye BCVA at pathway entry was treated as a pragmatic indicator consistent with later access along the visual trajectory; the focus is therefore on vision status at pathway entry as a pragmatic timeliness proxy rather than waiting time per se.

Despite extensive work on cataract coverage and eCSC, peer-reviewed evidence on within-system equity gradients in timeliness at the point of first-eye surgery using routinely collected EHR data remains limited, particularly in rapidly modernizing middle-income health systems [4–6, 8, 18]. Moreover, much of the literature emphasizes population-level coverage/outcomes, cross-country benchmarking, or waiting time, and fewer studies provide an implementation-ready, EHR-derived indicator specification that can be embedded into routine audit and equity monitoring.

China provides an informative context to examine equity in timeliness of access. Service modernization, including expansion of ambulatory/day-surgery models in tertiary hospitals, suggests that in developed regions capacity constraints may be less determinative than historically [24]. Nevertheless, urban–rural disparities in cataract burden and surgical coverage persist; demand-side barriers to uptake remain documented; and residual out-of-pocket spending and differential insurance generosity may influence uptake and reimbursement [25–29].

Against this background, we evaluated whether better-eye best-corrected visual acuity at surgical-pathway entry, a routinely recorded electronic health record–derived proxy of timeliness, differs systematically across prespecified residence–insurance strata among patients undergoing first-eye cataract surgery. We analysed consecutive first-eye cataract surgery cases identified from routinely collected discharge registry data and linked preoperative electronic health record assessments at a high-volume tertiary hospital in Yangzhou, Jiangsu Province (August 2024–July 2025). Differences were assessed by residence (urban vs. rural) and insurance type (employee vs. resident). In general, employee insurance tends to offer higher reimbursement and lower cost-sharing than resident insurance, although benefit design varies by locality. By incorporating case-mix adjustment and accounting for within-centre surgeon practice patterns, we aimed to provide a replicable indicator specification and risk-adjustment approach for equity-oriented monitoring of timeliness in cataract care.

## Methods

### Study design and setting

This retrospective cohort study used routinely collected discharge registry and EHR data to examine equity gradients in vision status at entry to the surgical pathway among patients undergoing first-eye cataract surgery. The study was conducted at Northern Jiangsu People’s Hospital Affiliated to Yangzhou University (Yangzhou, Jiangsu Province, China), a high-volume tertiary eye care provider. The primary aim was equity-oriented service evaluation using an EHR-derived access-timing proxy (vision status at pathway entry), rather than assessment of surgical technique effectiveness or prescriptive acuity thresholds for clinical decision-making.

Discharge registry data captured age, sex, residence classification, insurance type, principal discharge diagnosis, procedure name/code, surgeon identifier, and admission/discharge dates. EHR data captured preoperative ophthalmic assessments (including bilateral BCVA) and structured diagnostic/procedural fields used to derive systemic comorbidity and ocular history.

### Participants and variables

Consecutive cataract surgeries performed between 1 August 2024 and 31 July 2025 were screened. Eligible cases were identified using a principal discharge diagnosis of cataract and confirmed by procedure name and/or procedure code. Included procedures comprised phacoemulsification and extracapsular/intracapsular cataract extraction; intraocular lens implantation was performed when applicable, as recorded in the procedure name/code (including extraction procedures when an intraocular lens was implanted). Exclusion criteria included combined procedures (e.g., cataract surgery with glaucoma surgery or pars plana vitrectomy), any recorded history of cataract surgery in the fellow eye, non-local residence, and self-pay status. The index event was first-eye cataract surgery during the study period. First-eye status was determined from admission documentation of fellow-eye cataract surgical history. Patients with a recorded history of non-cataract intraocular surgery in the fellow eye were retained because first-eye status was defined by the absence of fellow-eye cataract surgery; this was evaluated in a prespecified sensitivity analysis. Primary analyses were restricted to local insured residents with residence recorded as urban or rural.

The primary outcome was preoperative better-eye BCVA at pathway entry for first-eye cataract surgery, expressed in logMAR. Better-eye BCVA was defined as the better (lower) logMAR value between the operated and fellow eyes measured during the same preoperative assessment. The hospital operates a day-surgery pathway in which patients typically complete preoperative testing and admission registration first and then await operative scheduling/notification; the interval from registration to surgery is approximately 3–10 days. BCVA was therefore taken from the admission-registration visit and represents vision status at entry into the surgical pathway, serving as a pragmatic proxy for binocular functional reserve when patients reach surgery rather than a prescriptive threshold for decision-making.

As part of the standard admission-registration assessment, BCVA is routinely measured in both eyes by trained personnel using trial-lens refraction and Snellen charts. Visual acuity was recorded as decimal acuity in the electronic health record and converted to logMAR as − log10(decimal acuity). Qualitative acuities were mapped as counting fingers = 1.9, hand motion = 2.3, light perception = 2.7, and no light perception = 3.0 logMAR. Records indicating that visual acuity could not be assessed (e.g., uncooperative examination) were treated as missing. Better-eye BCVA was calculated only when BCVA was available for both eyes; otherwise, the outcome was treated as missing.

Equity strata were defined a priori using residence (urban vs. rural) and insurance type (employee vs. resident insurance), yielding four groups: Urban–Employee, Urban–Resident, Rural–Employee, and Rural–Resident. Insurance type was defined according to the category displayed when the patient’s medical insurance card was read at admission registration. These categories correspond to China’s two main basic medical insurance schemes: the Urban Employee Basic Medical Insurance (UEBMI), which is largely payroll-contribution–based for formally employed workers and retirees, and the Urban and Rural Resident Basic Medical Insurance (URRBMI), which is funded through per-capita contributions plus government subsidies for individuals not covered by UEBMI (e.g., children/students, unemployed individuals, and many informal-sector and rural residents). In general, UEBMI is considered more generous (e.g., higher reimbursement and lower cost-sharing) than URRBMI, although benefit design varies by locality. Urban/rural residence was adjudicated using both the address recorded on the national identification card and the patient’s self-reported current habitual residence at admission; when inconsistent, classification prioritised self-reported current habitual residence (main place of living during the past 6 months). The final residence category was recorded in the admission registration record.

Case-mix covariates were prespecified and included age and sex; systemic comorbidity burden categorised as 0, 1, 2, or ≥ 3, defined as the count of the following coded systemic diagnoses present (each counted once): diabetes, hypertension, immune disease, cardiovascular disease, and malignancy; and operated-eye ocular history captured as any pre-existing ocular comorbidity and any prior surgery in the operated eye based on structured EHR fields. Eight surgeons performed the included procedures; surgeon fixed effects were included to account for within-centre surgeon-level differences (e.g., case-mix selection, referral/assignment patterns, and practice style) that could influence vision status at pathway entry, rather than to model surgeon-specific waiting lists.

### Statistical analysis

Baseline characteristics were summarised overall and by residence–insurance group using median (IQR) for continuous variables and n (%) for categorical variables. Associations with better-eye BCVA were evaluated using linear regression with heteroskedasticity-robust standard errors (HC3). The primary multivariable model included residence–insurance group, age (per 10-year increase), sex, comorbidity category, operated-eye ocular comorbidity, prior operated-eye surgery, and surgeon fixed effects. Adjusted marginal means (predictive margins) and 95% confidence intervals (CIs) were derived from the fully adjusted model.

For interpretability, better-eye BCVA was additionally categorised a priori as early (≤ 0.30 logMAR), mid (0.30– < 0.70), and late (≥ 0.70). Multinomial logistic regression (reference: mid) included the same covariates and surgeon fixed effects; results were reported as relative risk ratios with 95% CIs using HC3 robust standard errors. Prespecified age-stratified analyses were reported for < 65, 65–74, 75–84, and ≥ 85 years, including adjusted marginal means and within-stratum category distributions. Effect modification by age was assessed by adding a residence–insurance group-by-age interaction term to the fully adjusted linear model and testing it using a joint Wald test. All tests were two-sided with α = 0.05. Analyses were conducted in Python (statsmodels). Because all eligible cases within a fixed period of routinely collected clinical data were analysed, an a priori power calculation was not performed; precision is presented via 95% CIs.

Analyses were conducted using complete cases. Records with missing better-eye BCVA at pathway entry (as defined above) were excluded from regression models. A prespecified sensitivity analysis excluded patients with a documented history of non-cataract intraocular surgery in the fellow eye. As an additional robustness check, the primary linear model was re-estimated using standard errors clustered by surgeon.

The study adhered to the Declaration of Helsinki. The Ethics Committee of Northern Jiangsu People’s Hospital Affiliated to Yangzhou University approved the study (Approval No. 2026ky053) with a waiver of informed consent due to the retrospective design and use of de-identified routinely collected data.

## Results

During the study window (1 August 2024 to 31 July 2025), 1627 consecutive patients undergoing first-eye cataract surgery were identified. After restricting to local residents with residence recorded as urban or rural, 1456 patients remained; 171 non-local patients were excluded. Among local residents, self-pay patients (n = 18) were excluded, yielding 1438 local insured patients in the prespecified 2 × 2 residence–insurance strata (urban/rural × employee/resident insurance). Bilateral preoperative BCVA at pathway entry (admission-registration assessment) was measured in 1437 of 1438 patients (99.9%). The remaining patient had missing BCVA in both eyes due to inability to cooperate with testing and was excluded from complete-case analyses; accordingly, the complete-case analytic cohort comprised 1437 patients. Thirty-six patients (2.5%) had a recorded history of non-cataract intraocular surgery in the fellow eye and were retained for primary analyses and evaluated in a prespecified sensitivity analysis excluding such cases.

Baseline characteristics are shown in Table 1. The median age was 70 years (IQR, 61–77), and 813 patients (56.6%) were female. The four residence–insurance strata comprised Urban–Employee (n = 850), Urban–Resident (n = 151), Rural–Employee (n = 97), and Rural–Resident (n = 339). Marked binocular asymmetry was observed at presentation, with a median inter-eye difference (worse-eye minus better-eye logMAR) of 0.30 (IQR, 0.12–0.78).

**Table 1:** Baseline characteristics of local insured residents undergoing first-eye cataract surgery, by residence–insurance group (complete-case N = 1437)

Characteristic	Overall		Urban–employee	Urban–resident		Rural–employee	Rural–resident
No. of patients	1437		850	151		97	339
Age, years, median [IQR]	70 [61, 77]		70 [61, 78]	70 [62, 76]		64 [56, 73]	71 [62, 75]
Female, n (%)	813 (56.6%)		449 (52.8%)	105 (69.5%)		48 (49.5%)	211 (62.2%)
*Comorbidity burden (count), n (%)*							
0	465 (32.4%)		278 (32.7%)	38 (25.2%)		41 (42.3%)	108 (31.9%)
1	507 (35.3%)		294 (34.6%)	60 (39.7%)		30 (30.9%)	123 (36.3%)
2	340 (23.7%)		197 (23.2%)	41 (27.2%)		21 (21.6%)	81 (23.9%)
≥ 3	125 (8.7%)		81 (9.5%)	12 (7.9%)		5 (5.2%)	27 (8.0%)
*Selected systemic comorbidities, n (%)*							
Diabetes	408 (28.4%)		252 (29.6%)	45 (29.8%)		23 (23.7%)	88 (26.0%)
Hypertension	713 (49.6%)		414 (48.7%)	92 (60.9%)		39 (40.2%)	168 (49.6%)
Cardiovascular disease	312 (21.7%)		189 (22.2%)	33 (21.9%)		13 (13.4%)	77 (22.7%)
Immune disease	42 (2.9%)		24 (2.8%)	4 (2.6%)		5 (5.2%)	9 (2.7%)
Malignancy (cancer/leukemia)	24 (1.7%)		12 (1.4%)	2 (1.3%)		2 (2.1%)	8 (2.4%)
*Operated-eye ocular history, n (%)*							
Any pre-existing ocular comorbidity	423 (29.4%)		261 (30.7%)	33 (21.9%)		29 (29.9%)	100 (29.5%)
Retinal disease	81 (5.6%)		53 (6.2%)	7 (4.6%)		6 (6.2%)	15 (4.4%)
Glaucoma	29 (2.0%)		15 (1.8%)	3 (2.0%)		6 (6.2%)	5 (1.5%)
Uveitis	14 (1.0%)		8 (0.9%)	3 (2.0%)		0 (0.0%)	3 (0.9%)
Diabetic eye disease	110 (7.7%)		60 (7.1%)	13 (8.6%)		9 (9.3%)	28 (8.3%)
High-myopia retinal/choroidal degeneration	123 (8.6%)		84 (9.9%)	8 (5.3%)		4 (4.1%)	27 (8.0%)
Other ocular comorbidities	89 (6.2%)		57 (6.7%)	2 (1.3%)		6 (6.2%)	24 (7.1%)
Any prior surgery in operated eye, n (%)	142 (9.9%)		71 (8.4%)	18 (11.9%)		16 (16.5%)	37 (10.9%)
*Preoperative BCVA (logMAR), median [IQR]*							
Operated eye	0.52 [0.40, 1.00]		0.52 [0.30, 1.00]	0.70 [0.40, 1.90]		0.70 [0.40, 1.30]	0.70 [0.40, 1.90]
Fellow eye	0.30 [0.10, 0.52]		0.22 [0.10, 0.40]	0.30 [0.15, 0.52]		0.30 [0.10, 0.52]	0.40 [0.15, 0.70]
Better eye	0.30 [0.10, 0.52]		0.22 [0.06, 0.40]	0.30 [0.13, 0.52]		0.22 [0.10, 0.40]	0.30 [0.15, 0.52]
Inter-eye difference (worse − better)	0.30 [0.12, 0.78]		0.30 [0.10, 0.70]	0.40 [0.12, 0.90]		0.30 [0.12, 0.73]	0.30 [0.10, 1.00]

Values are median [IQR] or n (%). BCVA was measured at admission registration (pathway entry) using trial-lens refraction and Snellen charts, recorded as decimal acuity and converted to logMAR as − log10(decimal acuity); qualitative acuities were mapped as counting fingers = 1.9, hand motion = 2.3, light perception = 2.7, and no light perception = 3.0 logMAR. Better-eye BCVA was defined as the lower logMAR of the two eyes; inter-eye difference was defined as worse-eye minus better-eye logMAR. One Urban–Resident patient had missing BCVA in both eyes due to inability to cooperate (“exam_uncooperative”) and was excluded

**Fig. 1:**
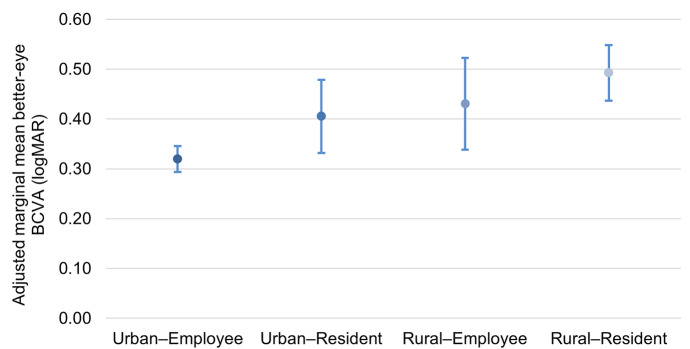
Adjusted marginal mean better-eye BCVA (logMAR) at pathway entry by residence–insurance group. Adjusted marginal means and 95% confidence intervals were derived from the fully adjusted linear regression model including surgeon fixed effects and HC3 heteroskedasticity-consistent robust standard errors (complete-case N = 1437). Higher logMAR indicates worse visual acuity. Group sizes are shown on the x-axis

Univariable and multivariable associations with better-eye BCVA at pathway entry are presented in Table 2. In the fully adjusted linear model, better-eye BCVA was worse (higher logMAR) in Urban–Resident (β = 0.09; 95% CI, 0.01–0.17; P = 0.034), Rural–Employee (β = 0.11; 95% CI, 0.01–0.21; P = 0.024), and Rural–Resident patients (β = 0.17; 95% CI, 0.11–0.24; P < 0.001), compared with Urban–Employee patients. Older age was associated with worse better-eye BCVA (β = 0.07 per 10-year increase; 95% CI, 0.04–0.10; P < 0.001), and any pre-existing ocular comorbidity in the operated eye was strongly associated with worse better-eye BCVA (β = 0.24; 95% CI, 0.17–0.30; P < 0.001). Any prior surgery in the operated eye was associated with slightly better better-eye BCVA (β =  − 0.10; 95% CI, − 0.19 to − 0.01; P = 0.044). Adjusted marginal means showed a graded pattern across strata (Fig. 1): 0.32 logMAR (Urban–Employee), 0.41 (Urban–Resident), 0.43 (Rural–Employee), and 0.49 (Rural–Resident).

**Table 2:** Factors associated with preoperative better-eye BCVA (logMAR) at pathway entry for first-eye cataract surgery (complete-case N = 1437)

Predictor	Unadjusted β (95% CI)	P	Adjusted β (95% CI)	P
Urban–Resident vs. Urban–Employee	0.08 (0.01–0.16)	0.042	0.09 (0.01–0.17)	0.034
Rural–Employee vs. Urban–Employee	0.07 (−0.03–0.16)	0.173	0.11 (0.01–0.21)	0.024
Rural–Resident vs. Urban–Employee	0.18 (0.12–0.24)	< 0.001	0.17 (0.11–0.24)	< 0.001
Age (per 10-year increase)	0.06 (0.03–0.08)	< 0.001	0.07 (0.04–0.10)	< 0.001
Female (vs. male)	0.03 (−0.02–0.08)	0.223	0.03 (−0.01–0.08)	0.181
Comorbidity count = 1 vs. 0	0.10 (0.04–0.15)	< 0.001	0.06 (0.01–0.12)	0.020
Comorbidity count = 2 vs. 0	0.12 (0.06–0.18)	< 0.001	0.08 (0.02–0.14)	0.007
Comorbidity count ≥ 3 vs. 0	0.15 (0.07–0.23)	< 0.001	0.08 (−0.01–0.16)	0.064
Any pre-existing ocular comorbidity (operated eye)	0.17 (0.11–0.22)	< 0.001	0.24 (0.17–0.30)	< 0.001
Any prior surgery in operated eye	0.02 (−0.06–0.10)	0.642	−0.10 (−0.19–−0.01)	0.044

Outcome was better-eye BCVA (logMAR), defined as the lower logMAR of the operated and fellow eyes measured at admission registration (pathway entry). Higher logMAR indicates worse vision. Unadjusted estimates are from separate univariable ordinary least squares models (one predictor per model). Adjusted estimates are from a multivariable model including residence–insurance group, age (per 10-year increase), sex, comorbidity count category (0/1/2/ ≥ 3), any pre-existing ocular comorbidity in the operated eye, any prior surgery in the operated eye, and surgeon fixed effects; heteroskedasticity-consistent robust standard errors were calculated using the HC3 estimator. Reference categories: Urban–Employee, male, comorbidity count = 0, and absence of the corresponding binary factor

**Fig. 2:**
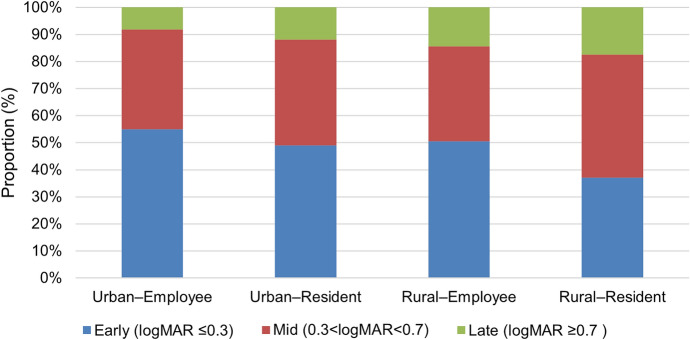
Distribution of better-eye BCVA categories at pathway entry across residence–insurance groups (complete-case N = 1437). Stacked bars show the within-group proportion of patients in each category. Better-eye BCVA was defined as the lower logMAR value of the operated and fellow eyes measured at admission registration (pathway entry). Categories were prespecified as early (logMAR ≤ 0.30), mid (0.30– < 0.70), and late (logMAR ≥ 0.70). Group sizes are shown on the x-axis

Using prespecified better-eye BCVA categories, 716 patients (49.8%) entered the surgical pathway in the early category (≤ 0.30 logMAR), 561 (39.0%) in the mid category (0.30– < 0.70), and 160 (11.1%) in the late category (≥ 0.70). The early category was most common among Urban–Employee patients (54.9%) and least common among Rural–Resident patients (37.2%), whereas the late category was most common among Rural–Resident patients (17.4%) (Fig. 2). In multinomial models (reference: mid), Rural–Resident patients were less likely to present in the early category (RRR = 0.52; 95% CI, 0.39–0.69; P < 0.001) and more likely to present in the late category (RRR = 1.76; 95% CI, 1.15–2.69; P = 0.009) than Urban–Employee patients; Rural–Employee patients also had a higher likelihood of presenting in the late category (RRR = 2.08; 95% CI, 1.02–4.25; P = 0.044) (Table 3).

**Table 3:** Multinomial logistic regression for better-eye BCVA categories at pathway entry (reference: Mid; complete-case N = 1437)

Predictor	RRR (Early vs. Mid)	95% CI	P	RRR (Late vs. Mid)	95% CI	P
Urban–Resident (vs. Urban–Employee)	0.81	0.55–1.19	0.284	1.47	0.78–2.78	0.235
Rural–Employee (vs. Urban–Employee)	0.74	0.45–1.19	0.214	2.08	1.02–4.25	0.044
Rural–Resident (vs. Urban–Employee)	0.52	0.39–0.69	< 0.001	1.76	1.15–2.69	0.009
Age (per 10-year increase)	0.66	0.58–0.75	< 0.001	1.14	0.90–1.44	0.266
Female (vs. male)	0.94	0.75–1.20	0.635	1.05	0.73–1.52	0.795
Comorbidity count = 1 vs. 0	0.93	0.70–1.24	0.620	1.21	0.74–1.97	0.452
Comorbidity count = 2 vs. 0	0.68	0.50–0.93	0.015	1.22	0.72–2.06	0.468
Comorbidity count ≥ 3 vs. 0	0.53	0.34–0.82	0.005	1.02	0.52–2.01	0.951
Any pre-existing ocular comorbidity (operated eye)	0.58	0.43–0.79	< 0.001	3.42	2.26–5.17	< 0.001
Any prior surgery in operated eye	1.29	0.82–2.04	0.275	0.97	0.53–1.80	0.935

Better-eye BCVA categories were prespecified as early (logMAR ≤ 0.30), mid (0.30– < 0.70), and late (≥ 0.70), using BCVA measured at admission registration (pathway entry). RRR indicates the adjusted relative risk ratio for early vs mid and late vs mid. Models included residence–insurance group, age (per 10-year increase), sex, comorbidity count category (0/1/2/ ≥ 3), any pre-existing ocular comorbidity in the operated eye, any prior surgery in the operated eye, and surgeon fixed effects; HC3 robust standard errors were used. Reference group: Urban–Employee

Age-stratified analyses showed that the residence–insurance gradient in better-eye BCVA at pathway entry persisted across age groups (Tables 4–5). Adjusted marginal mean better-eye BCVA worsened with increasing age and was consistently worst among Rural–Resident patients. For example, adjusted mean better-eye BCVA was 0.42 versus 0.24 logMAR (Rural–Resident vs. Urban–Employee) in those aged < 65 years, 0.50 vs. 0.32 logMAR in those aged 65–74 years, 0.55 vs. 0.38 logMAR in those aged 75–84 years, and 0.61 vs. 0.43 logMAR in those aged ≥ 85 years (Table 4). The proportion presenting in the late category increased steeply with age and was highest among Rural–Resident patients, reaching 52.9% among those aged ≥ 85 years (vs. 21.7% in Urban–Employee patients) (Table 5). In the full cohort, there was no evidence of effect modification by age (group-by-age interaction, joint Wald test P = 0.827).

**Table 4:** Age-stratified adjusted marginal mean better-eye BCVA (logMAR) at pathway entry for first-eye cataract surgery, by residence–insurance group

Age group	Urban–Employee	Urban–Resident	Rural–Employee	Rural–Resident
< 65	0.24 (0.21–0.28), n = 279	0.33 (0.25–0.41), n = 44	0.35 (0.26–0.45), n = 49	0.42 (0.35–0.48), n = 100
65–74	0.32 (0.30–0.35), n = 254	0.41 (0.34–0.48), n = 55	0.43 (0.34–0.53), n = 27	0.50 (0.44–0.55), n = 138
75–84	0.38 (0.34–0.41), n = 257	0.46 (0.39–0.54), n = 45	0.49 (0.39–0.59), n = 18	0.55 (0.49–0.61), n = 84
≥ 85	0.43 (0.38–0.49), n = 60	0.52 (0.43–0.61), n = 7	0.55 (0.44–0.65), n = 3	0.61 (0.54–0.68), n = 17

Adjusted marginal means and 95% CIs were derived from the fully adjusted linear model (including surgeon fixed effects) using BCVA measured at admission registration (pathway entry), with HC3 robust standard errors. Marginal means were estimated within each age stratum. Small cell sizes in some strata warrant cautious interpretation

**Table 5:** Age-stratified distribution of prespecified better-eye BCVA categories at pathway entry for first-eye cataract surgery, by residence–insurance group

Age group	Category	Urban–Employee n (%)	Urban–Resident n (%)	Rural–Employee n (%)	Rural–Resident n (%)
< 65					
	Early (≤ 0.30)	182 (65.2%)	26 (59.1%)	31 (63.3%)	50 (50.0%)
	Mid (0.30– < 0.70)	80 (28.7%)	14 (31.8%)	12 (24.5%)	31 (31.0%)
	Late (≥ 0.70)	17 (6.1%)	4 (9.1%)	6 (12.2%)	19 (19.0%)
65–74					
	Early (≤ 0.30)	152 (59.8%)	35 (63.6%)	11 (40.7%)	53 (38.4%)
	Mid (0.30– < 0.70)	82 (32.3%)	16 (29.1%)	10 (37.0%)	70 (50.7%)
	Late (≥ 0.70)	20 (7.9%)	4 (7.3%)	6 (22.2%)	15 (10.9%)
75–84					
	Early (≤ 0.30)	120 (46.7%)	13 (28.9%)	7 (38.9%)	22 (26.2%)
	Mid (0.30– < 0.70)	118 (45.9%)	24 (53.3%)	9 (50.0%)	46 (54.8%)
	Late (≥ 0.70)	19 (7.4%)	8 (17.8%)	2 (11.1%)	16 (19.0%)
≥ 85					
	Early (≤ 0.30)	13 (21.7%)	0 (0.0%)	0 (0.0%)	1 (5.9%)
	Mid (0.30– < 0.70)	34 (56.7%)	5 (71.4%)	3 (100.0%)	7 (41.2%)
	Late (≥ 0.70)	13 (21.7%)	2 (28.6%)	0 (0.0%)	9 (52.9%)

Values are n (% within each age group for each residence–insurance column). Better-eye BCVA categories were defined a priori as early (≤ 0.30 logMAR), mid (0.30– < 0.70), and late (≥ 0.70), using BCVA measured at admission registration (pathway entry). Small cell sizes in some strata warrant cautious interpretation

Results were robust in sensitivity analyses. When standard errors were clustered by surgeon, point estimates were unchanged with wider confidence intervals; the association for Rural–Resident patients remained statistically significant (Table S1). Excluding patients with a history of non-cataract intraocular surgery in the fellow eye (n = 36) yielded similar effect estimates in both the linear and multinomial models (Table S2). In secondary analyses of postoperative day-7 BCVA, follow-up completeness differed by strata, with lower completeness in Rural–Resident patients. Among patients with recorded day-7 BCVA, differences across strata were small; Urban–Resident patients had slightly worse day-7 BCVA, whereas estimates for Rural–Resident and Rural–Employee did not differ materially from Urban–Employee. Inverse probability weighted analyses accounting for differential day-7 BCVA recording yielded similar conclusions (Table S3).

## Discussion

In this real-world cohort from a high-volume tertiary centre in Eastern China, we observed clear, graded inequities in vision status at surgical-pathway entry for first-eye cataract surgery across prespecified residence–insurance strata. After adjustment for demographic factors, systemic comorbidity burden, operated-eye ocular history, and surgeon fixed effects, patients in lower-access strata—most notably Rural–Resident patients—entered the pathway with worse preoperative better-eye BCVA. Compared with Urban–Employee patients, the adjusted difference was approximately 0.17 logMAR (around 1–2 Snellen lines), which is clinically meaningful and compatible with later entry along a binocular functional reserve trajectory. Findings were concordant when the timeliness proxy was analysed continuously and using prespecified categories and remained robust in sensitivity analyses, suggesting that within-centre surgeon practice variation alone did not account for the observed gradients.

These findings contribute a timeliness dimension to cataract service evaluation that is not captured by coverage- and outcome-focused metrics alone. Effective cataract surgical coverage integrates whether surgery occurs with whether it delivers good postoperative vision, thereby aligning monitoring with benefit and equity [4, 5]. However, systems—and strata within the same system—can achieve similar surgical volumes and short-term outcomes while differing substantially in the functional stage at which patients reach surgery. Timeliness is a core domain of health-care quality [9], and avoidable delay can prolong functional limitation and threaten independence, particularly in older adults [9–11]. In this study, worse better-eye BCVA at pathway entry reflects greater functional severity at the point patients enter the surgical pathway; this complements waiting-time metrics by capturing “when” access occurs along the visual trajectory in a form that is routinely available and more readily standardised across EHR systems than timestamps that may be inconsistently defined or incomplete [6–8].

The age-stratified analyses underscore the clinical relevance of this timeliness perspective in an ageing population. Better-eye BCVA at pathway entry worsened with increasing age, and the residence–insurance gradient persisted across age groups. Although the oldest patients carried the greatest absolute burden of late-category entry, there was no evidence of effect modification by age, suggesting broadly parallel inequities across the age spectrum rather than disparities confined to a single age band. The concentration of late-category entry among the oldest Rural–Resident patients is particularly concerning because older adults may be more vulnerable to the consequences of prolonged visual impairment. Prior evidence indicates that waiting for cataract surgery is associated with reduced quality of life and further vision loss during the waiting period [12], and first-eye cataract surgery has been associated with reductions in falls in older adults in trials and meta-analyses [13, 14]. Although downstream functional outcomes were not measured here, systematic differences in binocular reserve at pathway entry are therefore directly relevant to patient-centred cataract care.

The observed gradients align with broader evidence that preoperative acuity at cataract surgery is system-sensitive rather than biologically fixed. Registry studies and cross-country comparisons show substantial variation and temporal shifts in preoperative better-eye acuity distributions, implying that health-system context and service organisation influence when patients reach surgery [16–18, 20]. Importantly, equity gradients have been documented even in mature systems, including worse impairment at first-eye surgery among more deprived groups and inequities in waiting times [21–23]. Our findings extend this literature by demonstrating within-centre equity gradients in a rapidly modernising, high-volume delivery model in China—an environment in which capacity constraints may be less determinative than historically [24]. In such settings, persistent residence–insurance gradients are compatible with upstream access processes and financial frictions remaining influential in shaping “revealed” timing of entry to surgery.

Several non-mutually exclusive mechanisms could plausibly contribute. Demand-side barriers may delay presentation, particularly while the better-seeing eye remains functional, consistent with evidence from rural China on perceived need, awareness, and barriers to uptake [26, 27]. Pathway frictions—geographic accessibility, referral processes, navigation burdens, and scheduling logistics—may persist even as ambulatory/day-surgery models expand throughput [24]. Financial frictions may also influence timing. Because UEBMI and URRBMI generally differ in reimbursement generosity and cost-sharing (with local variation), affordability-related barriers could plausibly delay pathway entry. However, insurance type may also proxy broader socioeconomic circumstances, and we lacked claims-level payment data to test mediation [28–31]. While this dataset did not capture referral source, travel distance, waiting-time timestamps, or reimbursement/out-of-pocket payments, the graded ordering across residence–insurance strata is compatible with a combined influence of demand, pathway, and financing factors. These mechanisms are testable in future work through linkage of EHR data with claims, scheduling timestamps, and geographic measures [8].

Operated-eye ocular comorbidity was strongly associated with worse better-eye BCVA at pathway entry and greater likelihood of late-category entry in adjusted analyses. This likely reflects non-cataract visual impairment and more complex care pathways rather than a pure access-timing effect. Notably, the residence–insurance gradient persisted after adjusting for ocular comorbidity, suggesting that measured ocular case mix alone does not explain the observed inequities.

Interpretation of “earlier” versus “later” access requires nuance. Cataract surgery should remain individualised and function-driven rather than governed by a single acuity threshold [15]. Accordingly, the aim was not to propose prescriptive BCVA cut-offs for surgery, but to evaluate whether a routinely captured EHR measure can serve as an equity-oriented timeliness proxy for monitoring. From an implementation perspective, routine reporting of equity-stratified distributions of better-eye BCVA at pathway entry—such as adjusted mean logMAR and the proportion entering in a late category—could complement coverage and postoperative outcome metrics by making timeliness inequities visible and enabling targeted improvement initiatives focused on earlier access among disadvantaged groups [4, 5].

This study has several strengths for pragmatic service evaluation. It used a consecutive cohort over a fixed one-year window, explicitly operationalised first-eye status at admission, and applied a binocularly anchored outcome aligned with everyday functioning. Primary-outcome availability was near-complete, and surgeon fixed effects helped account for within-centre practice variation.

These strengths support the internal validity of the observed gradients; however, several limitations should be considered when interpreting generalizability and mechanisms. First, the analysis was restricted to treated patients at a single tertiary centre and cannot quantify unmet need among individuals who never reached surgery; the findings therefore reflect inequities at the point of access within this delivery model rather than population-level disparities. Second, the timeliness proxy was anchored to the admission-registration visit; in a day-surgery pathway with surgery typically occurring within 3–10 days, it is best interpreted as functional severity at pathway entry rather than vision status on the day of surgery. Third, residence classification may remain imperfect despite adjudication because administrative address and self-reported habitual residence may not fully capture peri-urban boundaries or recent migration; any residual misclassification would likely be non-differential and attenuate between-group differences. Fourth, routine Snellen-based BCVA measurement and logMAR conversion introduce measurement variability that would also tend to bias associations toward the null. Finally, residual confounding by unmeasured socioeconomic factors, cataract morphology/severity, and pathway characteristics remains possible. In secondary analyses, differential completeness of postoperative day-7 recording across strata also highlights the need to address follow-up bias when outcome metrics are used for equity benchmarking.

Future work should replicate these findings across multiple centres and levels of care and link EHR data with claims and pathway timestamps to strengthen interpretability and policy relevance [8]. Integrating reimbursement and out-of-pocket payments, referral and scheduling dates, and measures of geographic accessibility would help disentangle demand-side barriers from pathway and financial frictions that may delay entry to surgery. Validation of BCVA-based timeliness indicators against patient-centred outcomes—such as self-reported visual function, mobility, and falls—would further support incorporating timeliness into routine cataract service dashboards [11, 13]. Collectively, these results support equity-stratified monitoring of better-eye BCVA at pathway entry as a scalable EHR-derived timeliness proxy that complements coverage and outcome metrics without implying a prescriptive surgical acuity threshold [4, 5, 15].

## Conclusions

In a high-volume tertiary eye-care setting in Eastern China, rural residence and resident insurance were associated with worse preoperative better-eye BCVA at surgical-pathway entry for first-eye cataract surgery, compatible with later access along the functional visual trajectory. Better-eye BCVA at pathway entry is a low-cost, routinely recorded EHR-derived indicator that may complement coverage and postoperative outcome metrics by adding a scalable timeliness dimension to cataract service evaluation. Equity-stratified reporting of this indicator, supported by case-mix adjustment and accounting for surgeon practice patterns, could help identify potentially modifiable pathway and financing frictions and inform targeted quality-improvement efforts.

## Supplementary Information

Below is the link to the electronic supplementary material.

Supplementary file1 (DOCX 26 KB)

## Data Availability

The datasets analysed in this study are not publicly available at the time of submission due to institutional data governance requirements and patient privacy protections. Upon acceptance, a fully de-identified dataset will be made publicly available as a Supplementary File alongside the published article.

## Abbreviations

BCVA: Best-corrected visual acuity
CI: Confidence interval
CF: Counting fingers
CSC: Cataract surgical coverage
eCSC: Effective cataract surgical coverage
HER: Electronic health record(s)
HC3: Heteroskedasticity-consistent standard errors (HC3 estimator)
HM: Hand motion
IQR: Interquartile range
LogMAR: Logarithm of the minimum angle of resolution
LP: Light perception
NLP: No light perception
UEBMI: Urban Employee Basic Medical Insurance
URRBMI: Urban and Rural Resident Basic Medical Insurance
RRR: Relative risk ratio
