# Supplementary material for: Residence and insurance inequities in the timeliness of access to first-eye cataract surgery in Eastern China
Source: Int Ophthalmol. 2026 Apr 6;46(1):193. doi: 10.1007/s10792-026-04069-9 (PMC13053540; doi:10.1007/s10792-026-04069-9)
Supplement: Supplementary file 1 — Supplementary file1 (DOCX 26 KB) [file 10792_2026_4069_MOESM1_ESM.docx]

**Table S1. Factors associated with better-eye BCVA (logMAR) at pathway entry for first-eye cataract surgery: standard errors clustered by surgeon (complete-case N=1,437).**

| Predictor | β (95% CI) | P |
| --- | --- | --- |
| Urban–Resident vs Urban–Employee | 0.08 (−0.04–0.21) | 0.185 |
| Rural–Employee vs Urban–Employee | 0.11 (−0.01–0.23) | 0.064 |
| Rural–Resident vs Urban–Employee | 0.17 (0.09–0.25) | <0.001 |
| Age (per 10-year increase) | 0.07 (0.04–0.10) | <0.001 |
| Female (vs male) | 0.03 (−0.02–0.07) | 0.223 |
| Comorbidity count = 1 vs 0 | 0.06 (−0.02–0.15) | 0.161 |
| Comorbidity count = 2 vs 0 | 0.08 (0.01–0.14) | 0.024 |
| Comorbidity count ≥3 vs 0 | 0.08 (−0.01–0.18) | 0.077 |
| Any pre-existing ocular comorbidity (operated eye) | 0.23 (0.17–0.29) | <0.001 |
| Any prior surgery in operated eye | −0.10 (−0.18–−0.01) | 0.031 |

**Notes:** Outcome was better-eye BCVA (logMAR), defined as the lower logMAR value of the operated and fellow eyes measured at admission registration (pathway entry). Higher logMAR indicates worse vision. Ordinary least squares regression included residence–insurance group, age (per 10-year increase), sex, comorbidity count category (0/1/2/≥3), any pre-existing ocular comorbidity in the operated eye, any prior surgery in the operated eye, and surgeon fixed effects. Standard errors were clustered by surgeon. All confidence intervals are 95%. Reference categories: Urban–Employee for residence–insurance group; male for sex; comorbidity count=0; no pre-existing ocular comorbidity in the operated eye; and no prior surgery in the operated eye. Age was modelled per 10-year increase.

**Table S2. Sensitivity analysis excluding patients with prior non-cataract intraocular surgery in the fellow eye (excluded n=36; analysed n=1,401).**

**Panel A. Continuous outcome: better-eye BCVA (logMAR) at pathway entry**

| Predictor | β (95% CI) | P |
| --- | --- | --- |
| Urban–Resident vs Urban–Employee | 0.09 (0.01–0.18) | 0.030 |
| Rural–Employee vs Urban–Employee | 0.10 (0.01–0.19) | 0.033 |
| Rural–Resident vs Urban–Employee | 0.18 (0.11–0.24) | <0.001 |
| Age (per 10-year increase) | 0.07 (0.04–0.09) | <0.001 |
| Female (vs male) | 0.02 (−0.02–0.07) | 0.314 |
| Comorbidity count = 1 vs 0 | 0.06 (0.00–0.11) | 0.040 |
| Comorbidity count = 2 vs 0 | 0.07 (0.02–0.13) | 0.013 |
| Comorbidity count ≥3 vs 0 | 0.09 (0.01–0.18) | 0.028 |
| Any pre-existing ocular comorbidity (operated eye) | 0.21 (0.14–0.27) | <0.001 |
| Any prior surgery in operated eye | −0.09 (−0.18–0.01) | 0.071 |

**Panel B. Multinomial outcome: early vs mid and late vs mid (better-eye BCVA categories at pathway entry)**

| Predictor | RRR (Early vs Mid) | 95% CI | P | RRR (Late vs Mid) | 95% CI | P |
| --- | --- | --- | --- | --- | --- | --- |
| Urban–Resident vs Urban–Employee | 0.87 | 0.58–1.30 | 0.497 | 1.82 | 0.96–3.46 | 0.067 |
| Rural–Employee vs Urban–Employee | 0.71 | 0.43–1.15 | 0.163 | 2.05 | 0.99–4.24 | 0.054 |
| Rural–Resident vs Urban–Employee | 0.53 | 0.40–0.71 | <0.001 | 1.94 | 1.26–3.00 | 0.003 |
| Age (per 10-year increase) | 0.66 | 0.58–0.75 | <0.001 | 1.11 | 0.88–1.41 | 0.389 |
| Female (vs male) | 0.95 | 0.75–1.20 | 0.646 | 1.00 | 0.68–1.45 | 0.985 |
| Comorbidity count = 1 vs 0 | 0.97 | 0.73–1.29 | 0.836 | 1.25 | 0.73–2.15 | 0.417 |
| Comorbidity count = 2 vs 0 | 0.65 | 0.48–0.89 | 0.007 | 1.07 | 0.63–1.82 | 0.797 |
| Comorbidity count ≥3 vs 0 | 0.43 | 0.26–0.72 | 0.001 | 1.00 | 0.48–2.08 | 0.997 |
| Any pre-existing ocular comorbidity (operated eye) | 0.62 | 0.45–0.84 | 0.002 | 3.25 | 2.13–4.95 | <0.001 |
| Any prior surgery in operated eye | 1.30 | 0.81–2.10 | 0.277 | 1.00 | 0.52–1.91 | 0.995 |

**Notes:** This prespecified sensitivity analysis excluded patients with a recorded history of non-cataract intraocular surgery in the fellow eye (excluded n=36; analysed n=1,401). Better-eye BCVA (logMAR) was defined as the lower logMAR value of the operated and fellow eyes measured at admission registration (pathway entry). Panel A reports ordinary least squares regression for continuous better-eye BCVA including surgeon fixed effects with HC3 robust standard errors. Panel B reports multinomial logistic regression for prespecified better-eye BCVA categories (reference: mid) including surgeon fixed effects with HC3 robust standard errors. Categories were defined a priori as early (logMAR ≤0.30), mid (0.30–<0.70), and late (logMAR ≥0.70). RRR indicates the adjusted relative risk ratio for early vs mid and late vs mid. Reference group: Urban–Employee. All confidence intervals are 95%.

**Table S3. Postoperative day-7 operated-eye BCVA availability and outcomes by residence–insurance group (local insured first-eye cataract surgery patients).**

**Panel A. Availability of postoperative day-7 operated-eye BCVA and adjusted odds of having a recorded day-7 BCVA (N=1,437).**

| Group | Day-7 CDVA available, n/N (%) | Adjusted OR (95% CI) | P |
| --- | --- | --- | --- |
| Urban–Employee | 816/850 (96.0%) | 1.00 (ref) | — |
| Urban–Resident | 144/151 (95.4%) | 0.83 (0.34–2.02) | 0.676 |
| Rural–Employee | 93/97 (95.9%) | 0.90 (0.27–2.95) | 0.864 |
| Rural–Resident | 311/339 (91.7%) | 0.48 (0.29–0.80) | 0.005 |

**Panel B. Factors associated with postoperative day-7 operated-eye BCVA (logMAR) among patients with available day-7 BCVA (n=1,364).**

| Predictor | β (95% CI) | P |
| --- | --- | --- |
| Urban–Resident vs Urban–Employee | 0.07 (0.00–0.14) | 0.035 |
| Rural–Employee vs Urban–Employee | 0.04 (-0.01–0.10) | 0.152 |
| Rural–Resident vs Urban–Employee | 0.02 (-0.01–0.06) | 0.225 |
| Preoperative CDVA (operated eye), per 1.0 logMAR worse | 0.19 (0.16–0.23) | <0.001 |
| Age, per 10-year increase | 0.05 (0.03–0.07) | <0.001 |
| Female (vs male) | 0.00 (-0.03–0.04) | 0.840 |
| Comorbidity count = 1 vs 0 | 0.03 (-0.03–0.09) | 0.300 |
| Comorbidity count = 2 vs 0 | 0.04 (-0.02–0.11) | 0.186 |
| Comorbidity count ≥3 vs 0 | 0.03 (-0.06–0.12) | 0.517 |
| Any pre-existing ocular comorbidity (operated eye) | 0.25 (0.20–0.30) | <0.001 |
| Any prior surgery in operated eye | 0.05 (-0.03–0.13) | 0.234 |
| Posterior capsule rupture (PCR) | 0.08 (-0.02–0.18) | 0.119 |

**Panel C. Inverse probability weighted analysis of postoperative day-7 operated-eye BCVA accounting for differential recording (n=1,364; weighted to N=1,437).**

| Predictor | β (95% CI) | P |
| --- | --- | --- |
| Urban–Resident vs Urban–Employee | 0.07 (-0.00–0.14) | 0.050 |
| Rural–Employee vs Urban–Employee | 0.04 (-0.02–0.10) | 0.198 |
| Rural–Resident vs Urban–Employee | 0.02 (-0.02–0.06) | 0.267 |
| Preoperative CDVA (operated eye), per 1.0 logMAR worse | 0.19 (0.16–0.23) | <0.001 |
| Age, per 10-year increase | 0.05 (0.03–0.07) | <0.001 |
| Female (vs male) | -0.02 (-0.05–0.02) | 0.345 |
| Comorbidity count = 1 vs 0 | 0.03 (-0.03–0.09) | 0.292 |
| Comorbidity count = 2 vs 0 | 0.04 (-0.02–0.11) | 0.205 |
| Comorbidity count ≥3 vs 0 | 0.03 (-0.06–0.12) | 0.529 |
| Any pre-existing ocular comorbidity (operated eye) | 0.25 (0.20–0.31) | <0.001 |
| Any prior surgery in operated eye | 0.05 (-0.04–0.14) | 0.256 |
| Posterior capsule rupture (PCR) | 0.08 (-0.03–0.19) | 0.142 |

**Notes:** Postoperative day-7 operated-eye BCVA was recorded as decimal acuity and converted to logMAR as −log10(decimal acuity); qualitative acuities were mapped as counting fingers=1.9, hand motion=2.3, light perception=2.7, and no light perception=3.0 logMAR. Values recorded as not assessable (e.g., inability to cooperate) were treated as missing.
Panel A: Multivariable logistic regression for having a recorded day-7 BCVA; covariates included residence–insurance group, preoperative operated-eye BCVA (per 1.0 logMAR), age (per 10-year increase), sex, comorbidity category (0/1/2/≥3), operated-eye ocular comorbidity, prior operated-eye surgery, posterior capsule rupture (PCR), and surgeon fixed effects; HC3 robust standard errors.
Panel B: Ordinary least squares regression for day-7 operated-eye BCVA including the same covariates and surgeon fixed effects; HC3 robust standard errors.
Panel C: Stabilized inverse probability weights were derived from Panel A (weight = Pr[R=1]/Pr[R=1|X]) and applied in weighted least squares regression including the same covariates and surgeon fixed effects; HC3 robust standard errors. Positive β indicates worse day-7 BCVA. Weight truncation at the 1st/99th percentile was assessed as a sensitivity analysis with similar results.
